# Molecular insights into mechanisms underlying thermo-tolerance in tomato

**DOI:** 10.3389/fpls.2022.1040532

**Published:** 2022-10-25

**Authors:** Achuit K. Singh, Pallavi Mishra, Sarvesh Pratap Kashyap, Suhas G. Karkute, Prabhakar Mohan Singh, Nagendra Rai, Anant Bahadur, Tusar K. Behera

**Affiliations:** ^1^ Division of Crop Improvement, ICAR-Indian Institute of Vegetable Research, Varanasi, Uttar Pradesh, India; ^2^ Division of Crop Production, ICAR-Indian Institute of Vegetable Research, Varanasi, Uttar Pradesh, India

**Keywords:** tomato, heat stress, breeding, genomics, CRiSPR/Cas, epigenetic regulation, omics, phenomics

## Abstract

Plant productivity is being seriously compromised by climate-change-induced temperature extremities. Agriculture and food safety are threatened due to global warming, and in many cases the negative impacts have already begun. Heat stress leads to significant losses in yield due to changes in growth pattern, plant phonologies, sensitivity to pests, flowering, grain filling, maturity period shrinkage, and senescence. Tomato is the second most important vegetable crop. It is very sensitive to heat stress and thus, yield losses in tomato due to heat stress could affect food and nutritional security. Tomato plants respond to heat stress with a variety of cellular, physiological, and molecular responses, beginning with the early heat sensing, followed by signal transduction, antioxidant defense, osmolyte synthesis and regulated gene expression. Recent findings suggest that specific plant organs are extremely sensitive to heat compared to the entire plant, redirecting the research more towards generative tissues. This is because, during sexual reproduction, developing pollens are the most sensitive to heat. Often, just a few degrees of temperature elevation during pollen development can have a negative effect on crop production. Furthermore, recent research has discovered certain genetic and epigenetic mechanisms playing key role in thermo-tolerance and have defined new directions for tomato heat stress response (HSR). Present challenges are to increase the understanding of molecular mechanisms underlying HS, and to identify superior genotypes with more tolerance to extreme temperatures. Several metabolites, genes, heat shock factors (HSFs) and microRNAs work together to regulate the plant HSR. The present review provides an insight into molecular mechanisms of heat tolerance and current knowledge of genetic and epigenetic control of heat-tolerance in tomato for sustainable agriculture in the future. The information will significantly contribute to improve breeding programs for development of heat tolerant cultivars.

## Introduction

Crop plants during their entire period of growth, from germination to maturation are inevitably challenged to a drove of abiotic stress factors inflicting a serious threat to their productivity. Being sessile, most plants are often confronted with one or multiple stress factors at a same time. Abiotic stress factors comprise extremities of temperature, salinity, pH, and drought. Tomato crop, in particular, is more vulnerable to heat stress because of its ability to set fruits only at a specific temperature range (Janni et al., 2020). Different abiotic stress factors, either independently or in combination often have a universal commutual impact over the physiological, morphological, biochemical and molecular pathways inducing cellular damages to crops and adversely affecting the growth, productivity, and ultimately the yield of tomato (Zinn et al., 2010). Among different abiotic stress factors, drought and heat represents a common example of stress factors which not only occurs simultaneously but also perniciously impacts the overall growth and productivity (Jiang and Huang, 2001). Studies suggests that most of the plants subjected to combination of drought stress (DS) and heat stress (HS) exhibited significant detrimental effects compared to their individual occurrence (Fahad et al., 2017). Amid the upsurging global warming apprehending a major risk to agricultural productivity, high temperature has emerged as a major yield limiting stress and threat to global food security. Temperature fluctuations usually disturb the natural growth and reproduction of plants and could mutilate molecular interactions required for normal growth and development (Zhao et al., 2020). Extreme temperature has already resulted in a contravening impact over global agriculture productivity, and adversely affects crop plants in numerous ways leading to quality and yield related losses to the farmers (Janni et al., 2020).

Cultivated tomatoes (*Solanum lycopersicum* L.) are the members of Solanaceae family, which includes more than 3000 species from both the Old and New Worlds (Knapp, 2002). A wild relative of cultivated tomato species, the cherry tomato (*Solanum lycopersicum* var. *cerasiforme*), was first discovered in South America and Mexico (Bai and Lindhout, 2007). It is not uncommon to find a large diversity of genetic variation among tomato species, particularily among self-incompatible wild species such as *S. peruvianum* and *S. chilense*. This prompted a reassessment of tomato phylogeny and integration of the genus *Lycopersicon* into the *Solanum*, which now includes the only domesticated species, *S. lycopersicum* and other wild relatives (Peralta et al., 2006).

According to FAOSTAT, globally 186.821 million tonnes of tomatoes were produced on 5,051,983 hectares in 2020 (FAO, 2022). This wonder fruit is packed with powerful phytochemicals that protect the body against many chronic degenerative diseases. Tomatoes are good source of vitamins like retinol and ascorbic acid, phenolic compounds, α- and β- carotene, lycopene, as well as glycoalkaloids like tomatine. In tomatoes, it is observed that the phyto-constituents remain bioavailable even after routine cooking, making them even more advantageous for our health. Moreover, World horticultural production is led by tomato production, which ranks among the most promising commodities. In spite of this, tomato yields generally remain low due to high temperature. Temperatures are frequently high in some regions of the world, causing tomato crops to suffer a lot. Being a widely cultivated fruit crop, even a slight increase in temperature may result in extreme production losses at the global level (Ayenan et al., 2019).

**Figure 1:**
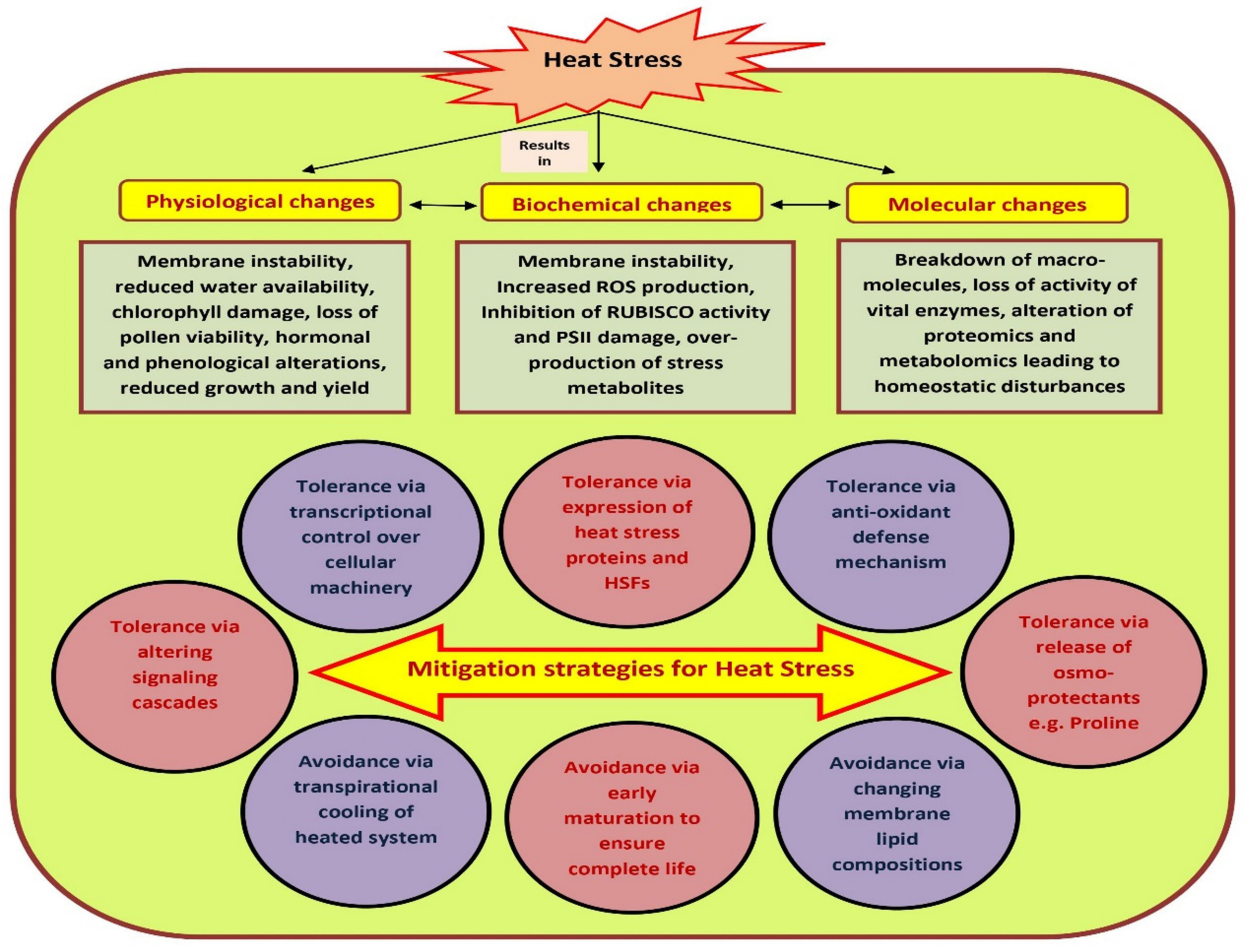
Heat stress impacts over plant physiological, biochemical, growth and yield. The effects of heat on plant growth can be seen on a morphological, physiological, metabolic as well as molecular level. However, plants have developed several mitigation strategies to combat the physio-morphological, biochemical and molecular changes posed due to heat stress. Under heat stress, plants’ immunity is also boosted. Plants can adopt ‘avoidance’ and ‘tolerance’ mechanisms to mitigate the challenges caused due to high temperature stress.

The susceptibility of tomato to HS is often a stage-specific phenomenon, and might vary from vegetative to reproductive stages of the same plant (Nievola et al., 2017). Many cultivated species may not reproduce successfully if they experience even a single hot day or cold night during the short period immediately following fertilization. The observed impact of HS also varies in different genotypes within species. In most of the crops including tomato, elevated temperature mainly interrupts with mechanisms concerned with the germination and viability of pollen grains (Zinn et al., 2010). To avoid such circumstances, plants have evolved certain in-built mechanisms to cope with the damaging effects of high temperature stress such as development of dehydrated embryos that could remain dormant within seeds for longer duration and development of dehydrated pollen grains within pollen tubes (Raja et al., 2019). In light of the fact that most of our food supply is the result of sexual reproduction in flowering plants, understanding how different plants handle stress during their reproductive or gametophytic phase is critical for managing the future of agricultural productivity (Zinn et al., 2010). Hence, to maximize agricultural productivity, it is necessary to identify weak links during sexual reproductive stages of plants. Heat stress tolerance may be defined as the competence of plants to survive and sustain usual growth and yield under high temperature (Janni et al., 2020). HS causes multi-dimensional and often irreversible damage to plants leading to unusual growth and development, reduced economic yield, and altered biochemical, morphological and physiological processes of plants such as early senescence, dying and scorching of leaves and stems, leaf abscission, sunburned aerial parts, growth inhibition, fruit discoloration and reduced quality etc. (Zhao et al., 2020) Though the aftermaths are many, HS generally have concomitant effects on shoot net assimilation rates and total dry weight of plants thereby leading to reduced plant productivity (**Figure 1**).

At elevated concentrations, HS causes reactive oxygen species (ROS) to be produced, which can cause severe oxidative damage to cells and possibly, death (Frank et al., 2009a). The increased ROS levels have been shown to play a role in triggering various transcriptional changes (Kotak et al., 2007). In addition to attenuating the effects of oxidative stress caused by abiotic stresses, ROS may also influence biotic stress-induced programmed cell death (PCD). In order to tailor the specific mechanisms to adapt to HS, the ROS could be coupled to other pathways such as Ca^2+^ signaling, kinase cascades, and hormone signals (Suzuki and Katano, 2018). As a consequence of HS, a large number of heat-shock proteins (HSPs) are produced, many of which are chaperones, and it has traditionally been suggested that protein denaturation causes HSP induction by HS (Scharf et al., 2012b). Recently, many HS sensors have been reported in several plants (Liu and Howell, 2016). Heat stress can also inhibit plant growth by affecting several molecular signaling pathways. The MAPK, ABF/bZIP, Ca2+-CBL-CIPK and CBF/DREB signaling pathways are among the most important signaling pathways involved in plant growth and acclimation to major abiotic stresses. Besides these, nitric oxide (NO) signaling can also be regarded as a very important aspect during stress responses in plants. Pollen-pistil interactions and pathogen defense have been shown to be influenced by NO signaling (Serrano et al., 2015). A pollen-derived NO have been reported to function to decrease the accumulation of H2O2 in papilla cells (Traverso et al., 2013; Serrano et al., 2015). Similarly, pollen tube elongation has also been reported to be influenced by the crosstalks between Ca^2+^ and NO signaling (Domingos et al., 2015). Therefore, in order to fully comprehend how plants respond to heat-stress, we must gain a deep understanding of how HS affects biochemical pathways, and decipher molecular pathways involved in heat-stress signaling.

Experimental studies on plants response to HS began in nineteenth century; however, analysis of cellular responses by HS induced alterations in the gene activities of polytene chromosomes of fruit fly *Drosophila* was the major breakthrough in this area (Ritossa, 1962). The study also exposed the highly conserved components of a general HS response mechanism which further laid the foundation for stress response studies throughout the prokaryotic and eukaryotic systems. However, until today even after over fifty years, uncovering various interconnected mechanisms leading to acquisition of thermo-tolerance in plants is a big challenge to the living world to achieve sustained growth and crop productivity under fast global climatic changes. Although the plants respond to HS in different ways, here we shall focus on molecular aspects and transcriptional reprogramming for counteracting the damage caused due to HS in crop plants. Recent advances in genome-wide analyses have revealed complex regulatory networks that control global gene expression, protein synthesis, chromatin modifications and metabolite composition under HS, which will be explored in the next section.

## Heat stress response in vegetative vs. reproductive stages

Breeding heat-tolerant crops is in high demand due to the insufficient adaptations to high temperatures in crop plants. The reproductive phase in flowering plants is one of the most vulnerable phases to heat stress; even one single hot day may bring about fatal effects on reproduction due to late flowering and inadequate seed set (Zinn et al., 2010). In tomato, heat stress tolerance is a quantitative trait. The study of sexual reproduction during stress conditions is quite challenging because the development of gametes and their fertilization take place rapidly but in a short time frame (Larkindale and Vierling, 2008). High temperature may lead to reduced fertility and flower abortion during inflorescence development (Luo, 2011). It has been found that high temperatures during meiosis and fertilization are associated with reduced fertility. Heat stress during flowering leads to a reduction in viable and germinating pollens in the male reproductive organs by reducing pollen development (Zinn et al., 2010). Several studies in tomato have shown that pollen development best starts at a temperature 21-25°C and enabled tomato plants to start fruit set (Kakani et al., 2005), but a higher temperature (above 29°C) caused male sterility due to insufficient pollen development (Frank et al., 2009b; Raja et al., 2019).

In contrast to male reproductive tissues, female gametophytes are more tolerant to high temperature stress (Driedonks et al., 2016). A temperature stress effect can be seen in the structure and function of corollas, carpels, stamens, as well as reduction in the number and size of floral organs (Morrison and Stewart, 2002). Overall, temperature stress may have a direct impact during the development of male and female gametes. Various reproductive stages during micro- and mega gametophyte development such as pollen germination, viability, growth, maturation, pollen tube growth, fertilization and embryo development are adversely affected by high temperature leading to improper fruit and seed development (Young et al., 2004; Raja et al., 2019).

Studies on tomato plant response to HS during vegetative and reproductive stages are expanding the horizons by availability of latest reports on the involvement of stage-specific transcriptomic studies and stress-regulated evaluations of gene expression during micro- and mega gametophyte development at transcriptional and post-translational levels (Keller et al., 2017). Besides the gene expressions, organ and stage-specific proteome profiles under HS have also been analyzed and reviewed in several crops including tomato (Kosová et al., 2011; Krasensky and Jonak, 2012). Furthermore, genetic engineering has demonstrated the significance of certain factors in conferring thermo-tolerance, and the accumulation of organ-specific proteins and metabolites during HS in heat-tolerant genotypes (Grover et al., 2013). A variety of post-translational modifications (PTMs) are also involved in adapting to HS. However, the correlation between transcripts, proteome and metabolites levels in heat stressed plant samples is often poor. This may be the result of alternative splicing, metabolite stability, compartmentalization and/or various post-transcriptional and post-translational modifications occurring inside plant cells (Hirayama and Shinozaki, 2010).

## Genetic regulation of tomato HSR

Researchers have long studied the physiological mechanisms behind HSR and thermo-tolerance (Prasad et al., 2017). However, the genetic mechanisms underlying plant HSR and identification of genes, QTLs and transcription factors (TFs) associated with heat stress tolerance in plants have advanced considerably in the recent years. Production of plants possessing HS-responsive genes *via* next-generation breeding strategies, and validating those plants on cutting-edge phenotyping platforms allows us to fully understand the role of HSR genes and develop more heat-tolerant cultivars (Janni et al., 2020). Generally, the genetic mechanisms regulating a plant’s response to high temperature stress are accompanied *via* physio-morphological acclimatization, acquisition of basal and acquired thermo-tolerance, modulation of plants immune response due to high temperature, and coordination of its biological circadian clock.

In tomatoes, remarkable harms can be caused by higher temperatures such as pre- and post-harvest, including burning of twigs and leaves, sunburn, stems, branches, abscission of leaves, preventing shoots and roots from developing, discoloration of fruit, and diminished production. Physio-morphological plant adaptations during high temperature stress mainly includes hypocotyl and petiole elongation, leaf hyponasty, and early flowering by promoting the accumulation of phytohormones such as auxins, gibberellin and brassinosteroids (Gray et al., 1998; Balasubramanian et al., 2006; Koini et al., 2009; Stavang et al., 2009). There are about 21 heat stress transcription factors (Hsfs) involved in tomato thermo-tolerance. There are two heat stress transcription factors in tomato, HsfA2 and HsfB1, but HsfA1 is the main controller of the heat shock response, as it regulates heat stress transcription at the transcriptional level (Scharf et al., 1998; Mishra et al., 2002). Specific interaction of HsfA1 and HsfA2 is reported to have synergistic activation of HSR in tomato (Chan-Schaminet et al., 2009).

As the ambient temperature rises, a tomato PIF4 gene playing a key role in acclimation to elevated environmental temperatures is transiently expressed and binds to the promoters of auxin biosynthesis genes in a temperature-dependent manner (Sun et al., 2012). Other than PIF4, MADS-box genes Short Vegetative Phase (SVP) and Flowering Locus M (FLM)/Mads Affecting Flowering (MAF) 1–5 also determine flowering time in response to temperature fluctuations (Balasubramanian et al., 2006; Lee et al., 2007).

Like any other organism, plants possess an inbuilt intrinsic mechanism of tolerance to HS known as basal thermo-tolerance, which is based on their inherent ability to cope with high temperature exposures and allow them to survive and acclimatize with the damaging effects of HS. Apart from this, they also possess ability to acquire certain characteristics later in their life cycle, which allows their sustained growth and economic development even in lethal episodes of HS conditions, and is referred as acquired thermo-tolerance. While certain transcriptional regulators and transcription factors (e.g. bHLH, DREB, WRKY and MAPK), heat acclimation proteins, and ROS detoxifying enzymes (such as catalases and peroxidases) are required only for basal thermo-tolerance (Vanderauwera et al., 2011), certain heat shock factors (HSFs) and molecular chaperones have been found compounded during acquired thermo-tolerance (Liu et al., 2011).

To confront the alarming effects of changing climate particularly global warming, land plants have been shown to constitutively express basal thermo-tolerance; while they also conditionally express an acquired thermo-tolerance for a short-period to confront more frequent and intense waves. While basal thermo-tolerance is an inherent property, acquired thermo-tolerance can also be artificially induced by exposing plants to a short duration of HS (for preparing it to accumulate and express high levels of transcripts; a stage called priming), followed by a recovery period.

In tomato plants, the circadian clock is a biological clock to integrate environmental cues such as light-dark photoperiod and temperature cycles with their own biological rhythms in a 24-hour period. According to studies, plant circadian clock consists of three interconnected transcriptional feedback loops namely a morning loop, an evening loop and a core oscillator loop (Hsu and Harmer, 2014). Circadian Clock-Associated1 (CCA1), Late Elongated Hypocotyl (LHY), and Timing of Cab Expression1/Pseudo-Response Regulator1 (TOC1/PRR1) are the three key regulators which control this complex network by inducing or repressing the activity of each other (Hsu and Harmer, 2014; Liu et al., 2015). Studies suggest that high temperature enhances CCA1 affinity for the oscillator genes, which is counteracted by casein kinase 2 and Flowering Basic Helix-Loop-Helix 1, thereby maintaining circadian rhythms (Portoles and Mas, 2010; Nagel et al., 2014). However, it remains elusive exactly how plants integrate circadian clocks with immunity under high temperatures.

High temperature has been shown to seriously affect plant immunity and has been well studied in the recent years (Hua, 2013). Temperature changes are often associated with other abiotic factors such as day light, night temperature and humidity. A most common effect of temperature is the inhibition of effector triggered immunity (ETI) by disrupting R-gene mediated resistance. ETI recognizes pathogen effectors by host proteins encoded by resistance (R) genes primarily consisting of nucleotide binding site-leucine rich repeats (NBS-LRR) class proteins (Martin et al., 2003). The first R-gene associated with high temperature mediated inhibition of resistance has been identified as SNC1 which in turn is negatively regulated by BONZAI1 (Yang and Hua, 2004; Zhu et al., 2010). Besides this, other negative regulators such as Bak1-Interacting Receptor-Like Kinase 1(BIR1), Bon1-Associated Protein 1 (BAP1), Constitutive Expresser of PR Genes 1(CPR1), Suppressor of Rps4-RLD (SRFR1) and MAP Kinase Phosphatase 1 (MKP1) (Yang and Hua, 2004; Gou and Hua, 2012) have also been identified. In addition, abscisic acid (ABA), salicylic acid (SA) and nitric oxide (NO) have also been shown to be involved in temperature mediated resistance inhibition. It will be fascinating to further elucidate the role of ABA, NO, and SA mediated defense responses under high temperatures.

## Molecular switches for plant heat stress response

Heat stress delimits economic yield of a healthy plant by affecting variety of physiological and biochemical activities such as cell growth and division, cell differentiation, respiration, photosynthesis, water transport, transpiration and nutrient uptake (Lippmann et al., 2019). Prolonged exposure of HS leads to production of extremely high amount of reactive oxygen species or ROS, which creates metabolic imbalances, actuates protein denaturation and deformation leading to perturbed membrane stability and loss of cellular integrity ultimately resulting in cellular stress (Hasanuzzaman et al., 2013; Hayes et al., 2021). Plants, therefore, need to confront these challenges to ensure their usual growth and development.

Being a multi-factorial trait, it would not be ideal to develop HS tolerance in crop plants using a single gene. Intensive studies on different crops have unveiled the involvement of a complex web of molecular switches, which are based on Transcription Factors, DNA-RNA, DNA-protein, RNA-protein and even protein-protein interactions that are responsive towards micro-molecules, endogenous metabolites, and external stimuli like stress (Haider et al., 2022). A deep understanding of mechanism and action and regulation of these molecular switches would assist plant breeders in not only identification of heat resistance genes, but also the connectedness of these molecular switches in regulating other cellular functioning and biochemical pathways, together regulating plant HS response in crop plants (Andrási et al., 2020).

Based on available literature and discoveries in the past, molecular switches for HS are usually explained by the studies on molecular chaperonins and heat shock factors (Qu et al., 2013). However, present is a changed scenario. A cognitive approach to the genetic control of plant response to high temperature stress has been accumulated beyond the involution of chaperons, phyto-hormones, secondary metabolites and other network pathways associated with synthesis of bio-active ingredients within plant. This approach relies on the participation of chromatin remodeling complexes, histone-sensors, covalent histone modifications, and heat stress transcription factor families within the nucleus, which shall be discussed in detail.

## Heat stress transcription factors/heat shock factors

Critical environmental conditions trigger plant responses through developmental, physiological, and biochemical mechanisms, which are in turn regulated by several transcription factors (TFs) (Mishra et al., 2002; Balyan et al., 2020). In response to HS, heat-shock genes are rapidly expressed, leading to accumulation of heat-shock proteins (HSPs), which are expressed and regulated by plant heat stress transcription factors or HSFs (Lin et al., 2011). The HSFs are crucial to plants’ response to abiotic stresses including HS by regulating the expression of many stress-responsive genes (Scharf et al., 2012a). Plant HSFs are now understood to play a role in individual or multiple abiotic stresses, especially in HS. In addition to stress responses, cell differentiation, proliferation and development are also responsible for regulating the expression of HSF in plants. First discovered in tomato (Czarnecka-Verner et al., 1995), the plant HSF families originate from complex superfamilies and are found in a diverse range of species such as *Arabidopsis*, rice, wheat, soybean, pepper with wheat and soybean having the maximum number of HSF genes (Baniwal et al., 2004; Scharf et al., 2012a; Xue et al., 2014; Fragkostefanakis et al., 2015; Guo et al., 2015). Besides, the plant HSFs family comprises of multiple HSF genes in their genome compared to eukaryotes. For example, *Caenorhabditis elegans*, *Drosophila melanogaster* and yeast genome possesses only a single HSF gene; however, *Arabidopsis* and rice genome composed of 20-25 HSFs genes (Guo et al., 2008). A number of HSFs have been identified and characterized in tomato in response to high temperatures (Scharf et al., 1990; Döring et al., 2000; Heerklotz et al., 2001; Yang et al., 2016).

It is observed that most plant HSFs are typically regulated by HS in the form of differential (up- or down) regulation of HSF genes. The expression of plant genes involved in the HSF pathway can also be influenced by other abiotic stresses such as cold, salinity, drought, and phyto-hormones such as salicylic acid, jasmonic acid, absscisic acid and ethylene. For example, HSFA2 and A6 transcripts became the predominant HSFs in wheat during HS, suggesting a regulatory role for these HSFs during HS (Xue et al., 2014). In tomato, HSFA1 and HSFA2 translocation from cytosol to nucleus is crucial to plant synergistic actions during HS and oxidative stress responses (Chan-Schaminet et al., 2009; Andrási et al., 2020). A large number of heat-responsive factors are negatively regulated by HSFBs, a Class B HSF which act as transcriptional repressors in tomato and *Arabidopsis* (Hahn et al., 2011; Arce et al., 2018; Zhuang et al., 2018; Haider et al., 2022).

The HSFs strategy can be efficiently used to create transgenic plants that are more tolerant to environmental stresses including HS (Andrási et al., 2020). However, there are many important points to consider. There is need for better understanding of HSF genes in plants, especially in important crop species such as tomato so as to minimize the negative effects in transgenic plants. Furthermore, due to functional divergence between HSF orthologs in different plant species, it is necessary to adapt and optimize the research focusing on HSFs function in both laboratory and field conditions and also beyond the model crops, to have a grasp over the regulatory mechanisms of HS-responsive HSF genes (Arce et al., 2018; Andrási et al., 2020). Further, marker-assisted selection can accelerate traditional crop breeding for stress tolerance traits. However, the selection of HSF genes as candidate genes and development of proper functional markers must be carefully considered due to HSFs being implicated in various developmental and stress response aspects (Haider et al., 2022).

## Epigenetic regulation of plant HSR: Understanding chromatin dynamics

Plants utilize a highly conserved epigenetic mechanisms to amend a better growth and development in order to confront variety of biotic and abiotic stresses (Saraswat et al., 2017; Benoit et al., 2019). This inbuilt-mechanism is associated with chromatin modifying strategy alterations of levels in gene expressions, and modulates plants inner plasticity to adapt themselves in adverse situations irrespective of the outer physiological changes (Gratani, 2014). Chromatin modifications is a prerequisite to an accurate transcription initiation and has been recognized as a significant mechanism facilitating plant normal growth under stress-challenged conditions. Chromatin is a highly condensed and tightly coiled structure composed of DNA and histone proteins. As a result of the tight coiling of chromatin, RNA polymerase and other transcription factors cannot access genes for transcription process. This compact structure must be opened for transcription to take place. This process is known as ‘chromatin remodeling’ and it allows transcription from an inactive state to an active one (Bannister and Kouzarides, 2011). Numerous biochemical changes in chromatin structure positively or negatively regulate gene activity, including DNA methylation.

Genetic evidence suggests that transgenerational adaptations to diverse stresses can be inherited across generations (Saraswat et al., 2017). However, only a limited number of studies have been conducted to validate this transmission of stress-induced changes in chromatin structure in plants. Plants are capable of modifying transcriptions in response to stress conditions by changing their chromatin structure, composition, and location, allowing them to maintain developmental and physiological changes over a long period of time (Pandey et al., 2016). Plants have also the ability to remember previous stresses and thus they can respond more efficiently whenever they are exposed to the stress again (Karkute et al., 2019). This phenomenon known as priming, is also associated with chromatin modification and often maintained independent of transcription process (Bäurle and Trindade, 2020). As a result of advances in high-throughput next-generation sequencing (NGS), well-assembled genome sequences, and antibodies to a plethora of DNA and histone modifications, studies of chromatin remodeling under variety of stresses have been greatly benefited (Kashyap et al., 2020; Zhao et al., 2020).

All eukaryotes contain chromatin packed into nucleosomes, which consists mainly of the histone family of proteins. Nucleosomes are repetitive units composed of 147 base pairs of DNA coiled around an octamer of H2A, H2B, H3, and H4 histones. Histone tails can be methylated at different amino acids and by different methods, including acetylation, methylation, ubiquitination, phosphorylation, ADP ribosylation, glycosylation, carbonylation, biotinylation and sumoylation. These modifications can either activate or repress transcription, respectively, by altering the chromatin’s configuration, thus controlling the accessibility of chromatin to transcriptional regulators (Eberharter and Becker, 2002; Li et al., 2007). Chromatin organization is accomplished mainly through enzymatic mechanisms in different ways: (i) utilizes chromatin remodelers that breaks the DNA-histone interaction *via* ATP hydrolysis, (ii) through DNA methylation, and (iii) covalent modifications of histone residues (Cedar and Bergman, 2009). In tomato, the interaction between HSFB1 and Histone acetyl transferase-1 (HAC1) regulates epigenetic expression in response to prolonged HS by recruiting histone acetyltransferase 1 (HAC1) (Bharti et al., 2004). Another study reports the upregulation of *SlyWRKY75* gene in tomato plants in response to abiotic stresses such as heat and drought. This mechanism is also under the epigenetic control (López-Galiano et al., 2018). In tomato, more than 80 WRKY transcription factors are reported to express in different developmental processes and under HS (López-Galiano et al., 2018).

### Chromatin remodeling complex

First identified in Arabidopsis, the SWItch/sucrose non-fermentable (SWI/SNF) complex is an important ATP-dependent chromatin remodeling complex critical for heat sensing, and requires ARP6 as an essential component for plant HSR (Liu et al., 2015). Overexpression of CHR12 (SNF2/Brahma-type chromatin-remodeling gene) inhibited the growth of primary stems and flower buds under heat and drought stress in *Arabidopsis*. On the other hand, Arabidopsis mutants lacking CHR12 exhibited reduced growth arrest compared to the wild-type plants (Mlynárová et al., 2007). These findings suggest that CHR12 mediates growth arrest under heat and drought conditions. In addition, upon temperature normalization, the H3-H4 chaperone CAF-1 (Chromatin Assembly Factor-1) is required for reloading nucleosomes onto chromosomes, suggesting its critical role in heat sensing and thermo-tolerance. Furthermore, HS not only induces HEAT-INTOLERANT 4 (HIT4) mediated decondensation of the chromocenter (Iwasaki and Paszkowski, 2014), but also leads to the rearrangement of complete 3-Dimensional genome structure in *Arabidopsis* indicating the importance of chromatin remodeling complexes in delineating the epigenetic prospect of plant HSR (Sun et al., 2020). However, there is little evidence of other ATP-dependent chromatin remodeling complexes roles in plant HSR.

### DNA methylation

DNA methylation occurs when DNA methyl transferases (DNMTs) incorporate a methyl group into the C-5 position of the cytosine ring of DNA. This process is epigenetic and completely heritable. Plant DNA is methylated in three sequence contexts: CG, CHG, and CHH (where H is A, T, or C) (Zhang et al., 2018). DNA methylation serves to guard the genome against selfish DNA elements, as well as to provide stability to the plant genome. DNA methylation is reported to be the first response to HS and plays a critical role in regulation of genes implicated in plant responses to high temperature (Li et al., 2016). DNA methylation facilitates the silencing of endogenous transposons and retrotransposons and thus facilitate genome stability (Chan et al., 2005). It has been shown that DNA methylation of promoter regions typically inhibits transcription initiation, but methylation within the gene body quantitatively slows transcription elongation in *Arabidopsis* (Zilberman et al., 2007). It is observed that DNA methylation changes under heat appear to vary from species to species without showing a consistent pattern. Only certain loci may experience methylation changes due to high temperature, and not all. High temperature increases methylation levels in some regions of the GUS but decreases them in others. In plants, DNA methylation is catalyzed by Domains Rearranged Methyl Transferase2 (DRM2), which in turn is regulated *via* RNA-directed DNA methylation (RdDM) pathway. It is therefore concluded that it is the RdDM pathway which is crucial for basal thermo-tolerance in plants (Popova et al., 2013). The role of DNA methylation in plant HSR is not clear, and it needs to be investigated whether DNA methylation regulates the plant circadian clock or basal immune response under heat stress.

### Histone covalent modifications

As described earlier, the histone proteins can be modified post-translationally through methylation, acetylation, ubiquitylation, phosphorylation and sumoylation. These modifications can change the amino acids exposed in the N-terminus tails of histones, changing the DNA histone interactions and blocking the protein binding sites (Kumar et al., 2021). Among several modifications, histone acetylation and methylation play an important role in the plant HSR, and are mediated *via* epigenetic regulators such as methyltransferases, acetyltransferases, demethylases and deacetylases (Kumar et al., 2021). Chromatin remodeling ATPases, along with methylated residues plays crucial roles in altering the position and composition of nucleosomes, thereby contributing to the basal thermo-tolerance in plants (Ohama et al., 2017). It is observed that gene silencing during plant exposure to heat occurs through DNA methylation *via* DNA methyl transferases and chromomethylases. H3K4 methylation (methylation of lysine 4 of histone 3) is commonly seen in facultative heterochromatins and exhibits a positive mark of transcription, but H3K9 methylation is a repressive mark of transcription, which is more common in constitutive heterochromatin. Similarly, histone acetyltransferases (HATs) acetylates histones and add a net negative charge to protein surfaces, reducing DNA interaction. Acetylation of histones facilitates transcription by loosening condensed chromatin (Kumar et al., 2021). On the other hand, removal of an acetyl moiety from histones by histone deacetylases (HDACs) facilitates chromatin condensation, indicating the significant roles of histone modifications in heat stress (Füßl et al., 2018; Kumar et al., 2021; Dar et al., 2022). Stress-induced histone modification has been observed in tomato for various kinds of stresses (Aiese Cigliano et al., 2013; González et al., 2013; Yu et al., 2018; Crespo-Salvador et al., 2020; Li et al., 2020; Wang et al., 2021).

### Histone sensors within nucleus

Several studies have shown the participation of histone sensors within nucleus as an epigenetic control for response to high temperature stress in plants. Histones are key components to maintain the genomic stability as well as functional integrity by altering the nucleosome configuration and their binding to different variants during the cell cycle. A study of Arabidopsis genome reveals the occurrence of around 13 H2A and 15 H3 histone proteins. The role of H3 histone proteins in heat sensing is not characterized. However, H2A genes have been well- characterized for their putative functions in regulating variety of plant stresses such as heat, drought and salinity. The well-known example is ARP6, a heat sensitive mutant gene known to mediate thermo-sensory responses in *Arabidopsis thaliana* by encoding SWI/SNF gene switch of the SWR1 complex. This complex is necessary for replacing the core histone protein H2A with an alternative protein dimer containing H2A.Z variants into the nucleosomes (Erkina et al., 2008; Clapier and Cairns, 2009; Erkina et al., 2010; Kumar and Wigge, 2010). The study clearly shows that mutants devoid of a functional ARP6 gene have reduced deposition of H2A.Z to the nucleosomes and affects transcriptional regulation of heat sensing machinery within the nucleus. Thus, ARP6 is an essential element for incorporation of H2A.Z variants in to the chromatin and mediating thermo-sensory responses in Arabidopsis.

## Small RNA−mediated HS responses

The small RNAs are non-protein-coding RNAs with an 18–30 nt length, that have emerged as important guide molecules in the control of gene expression. Small RNAs have been demonstrated to be involved in plant HS responses and play a vital role in temperature adjustments. In plants, the small RNA occur mainly in the form of small-interfering RNAs (siRNAs) and microRNAs (miRNAs), which differ in the proteins involved in their biogenesis and the mechanisms for their regulation (Ghildiyal and Zamore, 2009). In recent years, a growing body of research has shown that siRNA and miRNA play key roles in post-transcriptional control of plant growth by guiding specific mRNAs to degradation or by repressing translation. A number of studies have shown that miRNAs and endogenous siRNAs also play significant roles in plant temperature stress responses. The role of these RNAs in HS response has been confirmed in various model plants and crops. Not only this, the small RNAs are also involved in phytohormone signaling, the ROS-scavenging pathways, regulating the expression of plant HSFs/HSPs, as well as development of long-term HS memory in plants. In order to gain a deep understanding of small RNAs in thermal stress responses, it is necessary to explore how small RNAs regulate gene expression and how their regulatory mechanisms work.

### MicroRNAs

Plant miRNAs are 20-24 nt small RNAs that are derived from miRNA genes. Pol II transcribes miRNA genes into pri-miRNAs which are processed into stem-loop precursors called pre-miRNA, and then excised as either miRNA or miRNA duplex by endonuclease activity of the DCL1 protein complex. The miRNA matures and migrates to the cytoplasm, where they mediate transcriptional and post-transcriptional gene silencing (PTGS) through translational interference or splicing, and target chromatin for cytosine methylation (Rogers and Chen, 2013). Several miRNAs have been implicated in different aspects of plant growth and development, including temperature stress responses suggesting that these aspects may be linked *via* certain common mechanisms (Barrera-Figueroa et al., 2012). In addition, miRNA expression levels differ between different tissues and/or developmental stages under temperature stress but, only a few could be validated till date. There also have been a number of common heat-responsive miRNAs in different species such as miR156, 160, 167, 168, 169, 171, 395, 398, 408, and 827 families, but vary in their expression profiles as discovered by deep sequencing of small RNA and real-time PCR. For example, gene expression profiles of miR156 have been implicated to be induced by heat exposure in some species such as *Arabidopsis* and *Brassica rapa* (Yu et al., 2012; Stief et al., 2014), but repressed by similar conditions in case of rice (Sailaja et al., 2014). The *Arabidopsis* miR156 isoforms are highly induced by heat stress and target SQUAMOSA-PROMOTER BINDING-LIKE (SPL) transcription factor genes named SPL2 and SPL11, which are extremely critical for developmental transitions (Stief et al., 2014). A recent study demonstrated that miR156-SPL13 mediated HS response in alfalfa (Matthews et al., 2019). Another study showed that overexpression of soybean cytoplasmic male sterility (CMS)-based miR156b in *Arabidopsis* resulted in male sterility under HS (Ding et al., 2021). Unlike miR156, miR172 acts as a positive regulator of developmental transition by targeting members of the APETALA2 (AP2) family, because miR156 is highly expressed only in the juvenile phase, but miR172 is required for acquisition of adult stage (Ma et al., 2020). The miR172 overexpressing transgenic plants exhibited temperature insensitive early flowering (Lee et al., 2010). There is also evidence that miR172 functions in the thermo-sensory pathway to control ambient temperature-induced flowering under non-stress temperature conditions (Kouhi et al., 2020). These findings suggest that miRNA profiles are unique in closely related genotypes with different sensitivity to temperature and may be able to compound the heat sensitive regulators with other biotic and abiotic stresses as well.

Several studies on tomato miRNAs expressed in response to high temperature stress have been reported (Cardoso et al., 2018). Recently, 84 novel miRNAs were identified to be expressed during stigma exertion under high temperature stress in tomato (Pan et al., 2017). Several other unique miRNAs and their targets have been identified to respond to combined drought and heat stress in tomato (Shi et al., 2019; Zhou et al., 2020).

### Small-interfering RNAs

Heat stress is most threatening to the reproductive phase in the life cycle of flowering plants. Sometimes, even a single hot day can break a plant’s reproductive ability (Zinn et al., 2010). To protect the plants from damages caused due to HS, the plants siRNAs are produced from dsRNAs by DCL-activity, which directs TGS or target mRNA cleavage at homologous DNA loci using a silencing effector complex. Under high temperature stress, plant productivity is controlled by the target mRNA by regulating gene expression by the target mRNA, eliminating ROS generated due to temperature stress, and by controlling the phytohormone signaling pathway ultimately leading to HS tolerance in plants. Several endogenous siRNA classes have been discovered in Arabidopsis, including the endogenous derived siRNAs, trans-acting siRNAs, repeat-associated siRNAs, natural antisense transcript-derived siRNAs and double-strand-break-induced RNAs (Bologna and Voinnet, 2014). Similar to miRNAs, the endogenous siRNAs are also influenced by heat stress. However, the siRNAs identified for plant HS response are relatively fewer compared to the miRNAs. Previous studies have found that heat-induced retrotransposon called ONSEN accumulated in mutants with impaired siRNA biogenesis, indicating the possibility of siRNAs roles in HS responses (Ito et al., 2011). In *Arabidopsis*, a particular class of phasiRNAs and tasiRNAs was found to decrease significantly under heat stress, suggesting that they regulate HS responses (Li et al., 2014). Recently, it was shown that tasiRNA degradation due to HS has been implicated in transgenerational thermo-memory of early flowering and attenuated immunity by targeting HTT5, providing new insight into the impact of HS on reproductive fitness of the progeny (Liu et al., 2019). Recently, maize tassels and roots exposed to high temperatures exhibited a significant reduction in transposable element-derived siRNAs, while the nearby genes showed a tendency to become downregulated. However, the underlying mechanism still remains unpredictable (He et al., 2019).

## CRISPR technology for engineering thermo−tolerant crop plants

New plant breeding technologies have recently emerged as alternatives to genetically modified or GM crops as a means of speeding up the introduction of improved traits. In contrast to GM crops, precise genome-processing methodologies include clustered regularly interspaced short palindromic repeats (CRISPR)/CRISPR-associated proteins (Cas), transcription activator-like effector nucleases (TALENs), meganucleases and zinc-finger nucleases (ZFNs) based protocols (Pickar-Oliver and Gersbach, 2019). These technologies use site-specific nucleases which can be utilized in all-round aspects of plant breeding including gene knock-out, knock-in, gene pyramiding and *in-vitro* and targeted mutagenesis. Compared to conventional plant breeding approaches, which may take up to ten years to develop a variety, this technology is quick, precise and offer significant economic benefits (Chen et al., 2019).

The CRISPR/Cas technology has been widely adopted in plant developmental biology to identify genes and understand the molecular mechanisms of variety of traits. However, in the last few years, CRISPR technology has been substantially applied in agriculture for the development of abiotic stress-tolerant crops by targeting genes associated with plant sensitivity (S genes and cis-regulatory sequences) and tolerance (or T genes). In plants, the type II CRISPR/Cas9 and type V CRISPR/Cas12a from *Streptococcus pyogenes* and *Francisellano vicida*, respectively have been widely repurposed as genome editing systems (Xie and Yang, 2013; Bernabé‐Orts et al., 2019). In both systems, an RNA-dependent DNA endonuclease (Cas9 or Cas12a) operates in conjunction with an RNA molecule configuring specificity for target DNA. There have been reports of CRISPR/Cas9 and Cas12a mediated abiotic stress tolerance in several species such as Arabidopsis, wheat, rice, barley, millets and tomato. It is also reported that plants respond to a variety of environmental stresses by activating their mitogen-activated protein kinases or MAPKs (Matsuoka et al., 2018). Recently, this concept was deployed for CRISPR/Cas9 mediated editing of *SlMAPK3* gene in tomato for enhanced HS tolerance in cultivated tomato compared to wild type plants. The mutants not only exhibited HS tolerance but also showed decreased ROS content, less wilting, reduced membrane damage, increased antioxidant enzyme activities and elevated expressions of HSP genes and HSFs (Yu et al., 2019). A multiplex genome editing *via* CRISPR/Cas9 has been utilized to interrupt a hybrid proline-rich protein 1 (*SlHyPRP1*), a negative regulator of salt stress response in tomato. A later analysis revealed that precise elimination of *SlHyPRP1* functional domains resulted in high salinity tolerance in tomato (Tran et al., 2021).

The CRISPR-based strategy is not only limited to genome editing, but nowadays, it is also being utilized in emerging concepts like epigenome editing where the epigenetic modifiers are fluxed along with the Cas9 system to target DNA methylation *via* RNA-directed DNA methylation (RdDM) pathway. (Papikian et al., 2019). The dCas9-SunTag-VP64 system targeting SUPERMAN and FWA promoters for DRM2 methylation and N6-methyleadenosine (m6A) modification are the most frequently used types of regulatory mechanisms for epigenome editing in eukaryotes (Papikian et al., 2019; Yue et al., 2019). The CRISPR-based m6A editing system comprising m6A enzymes (writers or erasers) fused to the Cas13 protein has been recently demonstrated in *Arabidopsis*, tobacco, rice and tomato plants (Zheng et al., 2020; Zhou et al., 2022a). Thus, the present sketch reveals that this technology is proving to be a great asset to agriculture in transforming the development of crops to be more tolerant of biotic/abiotic stresses and climate change.

## Integrated ‘*OMICS’* approaches for developing thermo-tolerance in crop plants

**Figure 2:**
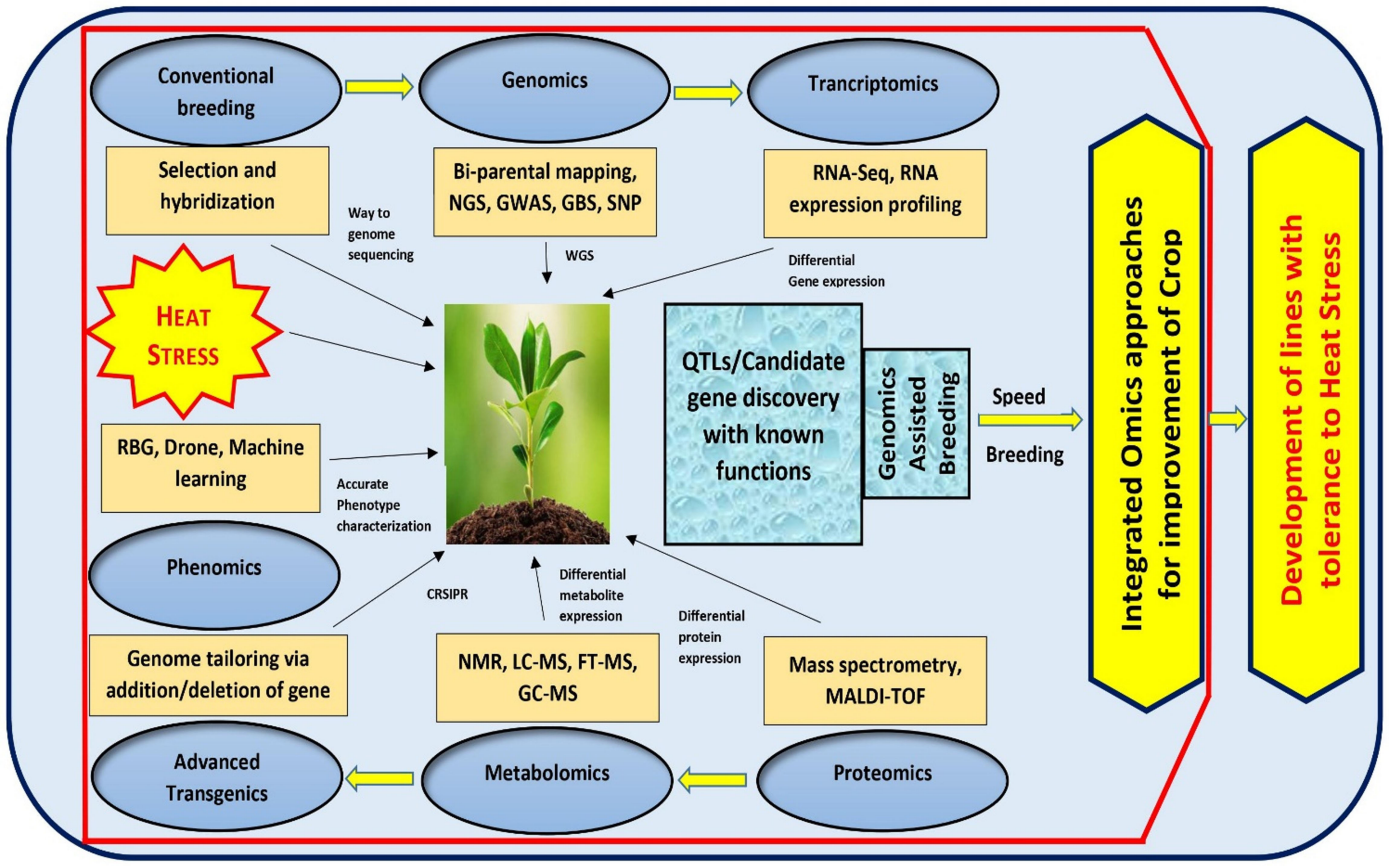
An integrated *‘Omics’* approach to improve tomato heat stress tolerance. By using bi-parental mating, GWAS mapping and Genotyping by Sequencing of large crop germplasm, genomic approaches can be implemented to shed light on the possible genes governing heat tolerance. The transcriptomic, proteomic, and metabolomics approaches, can be exploited to understand the functions of various genes and QTLs controlling heat tolerance. Additionally, tomato breeding could be improved through emerging speed breeding approaches.

To combat the challenge of climate change, it is imperative to focus more on the pre-breeding activities for introduction of novel alleles and new genetic variants into the breeding population that contribute to drought/heat tolerance. With advanced approaches such as QTL mapping, genome wide association studies, SNPs, GBS, NGS, WGS and WGRS approaches, it is possible to elucidate plethora of meaningful marker-trait associations, important gene variants and haplotypes across the genome conferring stress tolerance (Kilian et al., 2021). This can also lead to an in-depth understanding of the genetic makeup of plants, signaling cascades and their ability to adapt under multiple stress conditions. The identified genomic regions could be further investigated for stress tolerance in crop plants using the integrated ‘omics’ approaches, wherein, candidate genes are studied in detail *via* genomics, transcriptomics, proteomics, metabolomics and recently emerging artificial emergence (AI) based field phenotyping technology called phenomics (Bhardwaj et al., 2021; Zhou et al., 2022b). While the genomics approach can be used to discover the putative gene(s) conferring stress tolerance in plants, understanding the function of multiple genes and their complex networks controlling stress tolerance at the phenotypic level would greatly benefit from transcriptomic, proteomic and metabolomic approaches (Chaudhary et al., 2019). Emerging phenomics approaches with AI and machine learning shall facilitate precise phenotyping and improve the understanding of stress response in varying environmental conditions (Basavaraj and Rane, 2020). Moreover, new speed breeding techniques can also facilitate all round crop improvement. Parallel to these, transgenic and genome editing technology also supply us with the possibility of designing heat resistant crops by adding and/or deleting target DNA sequence within the genomic regions (Raza et al., 2021). The integration of these ‘omics’ approaches with conventional breeding strategies have resulted in enhanced crop performance under different stressed conditions including heat in several crops **(** **Figure 2** **)**. The omics revolution generates massive amounts of data, and sufficient advances have been achieved in computational tools for accurate analysis. In case of tomato, much progress has been achieved in genomics and transcriptomics to address various abiotic stresses, but major branches like metabolomics, proteomics, and phenomics are still lagging. Therefore, there is a need to focus on different omics tools and integrated approaches to have a deeper coverage of the entire genetic, functional and structural components and to successfully manage the conditions like heat stress (Raza et al., 2021; Taheri et al., 2022).

## Outlook

Being sessile, plants cannot avoid the myriad of environmental stresses to which they are exposed throughout their life span. Also, the growth and development of a plant is greatly dependent on the temperature of their environment. Therefore, a thorough understanding of mechanisms how plants respond to environmental stresses like drought, salinity and heat is essential for improving their stress tolerance and productivity. In the world, tomato (*Solanum lycopersicum*) ranks second in terms of cultivation. As a result of HS, tomato yield is reduced, both vegetatively and reproductively. Therefore, there is an increasing demand for tomatoes in regions with high temperatures. A comparative transcriptomics approach in cultivated and contrasting cultivars has not yet been exploited to understand the effects of HS on tomato plants. In order to gain a deeper understanding of HSR in tomato, the advanced techniques has to be applied. It is observed that the plants are able to adapt themselves to heat stress conditions by regulating gene expression, protein synthesis and metabolism. Expression of heat induced genes are primarily regulated by HSFs and HSPs which are key factors in acquisition of heat stress tolerance in tomato by binding to cis-acting elements in the promoter region of heat-induced genes. Besides the involvement of signaling cascades, ions, metabolites, ROS, HSFs and HSPs, many evidences of epigenetic and transgenerational memory inheritance have been reported when stressed plants are in the middle stage of sporophyte development. This suggests that the plants epigenetic landscape along DNA replication, repair, transcription, translation, gene splicing and PTGS mechanisms during heat exposure are needed to be decoded in detail. These days, the CRISPR/Cas-based tools are being developed in many crops for targeting genetic and epigenetic modifications. The versatility of this tool can be explored in the study of multiple stresses including heat stress in plants and could lead to identification of specific regulators controlling specific targets during heat exposure. There are enormous applications for genome and epigenome editing using CRISPR/Cas tools in fundamental research, and these techniques are now being tapped more and more for developing heat-tolerant ‘smart crops’. Meanwhile, the current obstacles in basic and applied plant research can be overcome by carrying out multidisciplinary ‘omics’tools in an integrated manner to have more access to the genetic makeup in detail. By successful implementation of these technologies, the anticipated challenges presented by climate change could be alleviated to many folds and nutritional requirements for humanity could be met.

## Author contributions

AKS, PM and SPK conceived the idea and wrote the manuscript. SPK, PMS, AB, NR, and TKB critically evaluated the manuscript. All authors contributed to the article and approved the submitted version.

## Funding

The work is supported under National Innovations in Climate Resilient Agriculture Project of Indian Council of Agricultural Research, India.

## Conflict of interest

The authors declare that the research was conducted in the absence of any commercial or financial relationships that could be construed as a potential conflict of interest.

## Publisher’s note

All claims expressed in this article are solely those of the authors and do not necessarily represent those of their affiliated organizations, or those of the publisher, the editors and the reviewers. Any product that may be evaluated in this article, or claim that may be made by its manufacturer, is not guaranteed or endorsed by the publisher.

## References

[B1] Aiese Cigliano R. Sanseverino W. Cremona G. Ercolano M. R. Conicella C. Consiglio F. M. (2013). Genome-wide analysis of histone modifiers in tomato: gaining an insight into their developmental roles. BMC Genomics 14, 1–20. doi: 10.1186/1471-2164-14-57 23356725 PMC3567966

[B2] Andrási N. Pettkó-Szandtner A. Szabados L. (2020). Diversity of plant heat shock factors: regulation, interactions, and functions. J. Exp. Bot. 72, 1558–1575. doi: 10.1093/jxb/eraa576 33277993

[B3] Arce D. Spetale F. Krsticevic F. Cacchiarelli P. Las Rivas J. D. Ponce S.. (2018). Regulatory motifs found in the small heat shock protein (sHSP) gene family in tomato. BMC Genomics 19, 860. doi: 10.1186/s12864-018-5190-z 30537925 PMC6288846

[B4] Ayenan M. Danquah A. Hanson P. Ampomah-Dwamena C. Sodedji F. Asante I. K.. (2019). Accelerating breeding for heat tolerance in tomato (Solanum lycopersicum l.): an integrated approach. Agronomy 9, 720. doi: 10.3390/agronomy9110720

[B5] Bai Y. Lindhout P. (2007). Domestication and breeding of tomatoes: what have we gained and what can we gain in the future? Ann. Bot. 100, 1085–1094. doi: 10.1093/aob/mcm150 17717024 PMC2759208

[B6] Balasubramanian S. Sureshkumar S. Lempe J. Weigel D. (2006). Potent induction of arabidopsis thaliana flowering by elevated growth temperature. PloS Genet. 2, e106. doi: 10.1371/journal.pgen.0020106 16839183 PMC1487179

[B7] Balyan S. Rao S. Jha S. Bansal C. Das J. R. Mathur S. (2020). Characterization of novel regulators for heat stress tolerance in tomato from Indian sub-continent. Plant Biotechnol. J. 18, 2118–2132. doi: 10.1111/pbi.13371 PMC7540533 32163647

[B8] Baniwal S. K. Bharti K. Chan K. Y. Fauth M. Ganguli A. Kotak S.. (2004). Heat stress response in plants: a complex game with chaperones and more than twenty heat stress transcription factors. J. Biosci. 29, 471–487. doi: 10.1007/BF02712120 15625403

[B9] Bannister A. Kouzarides T. (2011). Regulation of chromatin by histone modifications. Cell Res. 21, 381–395. doi: 10.1038/cr.2011.22 21321607 PMC3193420

[B10] Barrera-Figueroa B. E. Gao L. Wu Z. Zhou X. Zhu J. Jin H.. (2012). High throughput sequencing reveals novel and abiotic stress-regulated microRNAs in the inflorescences of rice. BMC Plant Biol. 12, 1–11. doi: 10.1186/1471-2229-12-132 22862743 PMC3431262

[B11] Basavaraj P. Rane J. (2020). Avenues to realize potential of phenomics to accelerate crop breeding for heat tolerance. Plant Physiol. Rep. 25, 594–610. doi: 10.1007/s40502-020-00552-2

[B12] Bäurle I. Trindade I. (2020). Chromatin regulation of somatic abiotic stress memory. J. Exp. Bot. 71, 5269–5279. doi: 10.1093/jxb/eraa098 32076719

[B13] Benoit M. Drost H.-G. Catoni M. Gouil Q. Lopez-Gomollon S. Baulcombe D.. (2019). Environmental and epigenetic regulation of rider retrotransposons in tomato. PloS Genet. 15, e1008370. doi: 10.1371/journal.pgen.1008370 31525177 PMC6762207

[B14] Bernabé-Orts J. M. Casas-Rodrigo I. Minguet E. G. Landolfi V. Garcia-Carpintero V. Gianoglio S.. (2019). Assessment of Cas12a-mediated gene editing efficiency in plants. Plant Biotechnol. J. 17, 1971–1984. doi: 10.1111/pbi.13113 30950179 PMC6737022

[B15] Bhardwaj A. Devi P. Chaudhary S. Rani A. Jha U. C. Kumar S.. (2021). ‘Omics’ approaches in developing combined drought and heat tolerance in food crops. Plant Cell Rep. 41, 699–739. doi: 10.1007/s00299-021-02742-0 34223931

[B16] Bharti K. Von Koskull-DoüRing P. Bharti S. Kumar P. Tintschl-KoüRbitzer A. Treuter E.. (2004). Tomato heat stress transcription factor HsfB1 represents a novel type of general transcription coactivator with a histone-like motif interacting with the plant CREB binding protein ortholog HAC1. Plant Cell 16, 1521–1535. doi: 10.1105/tpc.019927 15131252 PMC490043

[B17] Bologna N. G. Voinnet O. (2014). The diversity, biogenesis, and activities of endogenous silencing small RNAs in arabidopsis. Annu. Rev. Plant Biol. 65, 473–503. doi: 10.1146/annurev-arplant-050213-035728 24579988

[B18] Cardoso T. C. D. S. Alves T. C. Caneschi C. M. Santana D. D. R. G. Fernandes-Brum C. N. Reis G. L. D.. (2018). New insights into tomato microRNAs. Sci. Rep. 8, 1–22. doi: 10.1038/s41598-018-34202-3 30375421 PMC6207730

[B19] Cedar H. Bergman Y. (2009). Linking DNA methylation and histone modification: patterns and paradigms. Nat. Rev. Genet. 10, 295–304. doi: 10.1038/nrg2540 19308066

[B20] Chan S. W.-L. Henderson I. R. Jacobsen S. E. (2005). Gardening the genome: DNA methylation in arabidopsis thaliana. Nat. Rev. Genet. 6, 351–360. doi: 10.1038/nrg1601 15861207

[B21] Chan-Schaminet K. Y. Baniwal S. K. Bublak D. Nover L. Scharf K.-D. (2009). Specific interaction between tomato HsfA1 and HsfA2 creates hetero-oligomeric superactivator complexes for synergistic activation of heat stress gene expression. J. Biol. Chem. 284, 20848–20857. doi: 10.1074/jbc.M109.007336 19491106 PMC2742850

[B22] Chaudhary J. Khatri P. Singla P. Kumawat S. Kumari A. Vikram A.. (2019). Advances in omics approaches for abiotic stress tolerance in tomato. Biology 8, 90. doi: 10.3390/biology8040090 PMC6956103 31775241

[B23] Chen K. Wang Y. Zhang R. Zhang H. Gao C. (2019). CRISPR/Cas genome editing and precision plant breeding in agriculture. Annu. Rev. Plant Biol. 70, 667–697. doi: 10.1146/annurev-arplant-050718-100049 30835493

[B24] Clapier C. R. Cairns B. R. (2009). The biology of chromatin remodeling complexes. Annu. Rev. Biochem. 78, 273–304. doi: 10.1146/annurev.biochem.77.062706.153223 19355820

[B25] Crespo-Salvador Ó. Sánchez-Giménez L. López-Galiano M. Fernández-Crespo E. Scalschi L. García-Robles I.. (2020). The histone marks signature in exonic and intronic regions is relevant in early response of tomato genes to botrytis cinerea and in miRNA regulation. Plants 9, 300. doi: 10.3390/plants9030300 PMC7154849 32121544

[B26] Czarnecka-Verner E. Yuan C.-X. Fox P. C. Gurley W. B. (1995). Isolation and characterization of six heat shock transcription factor cDNA clones from soybean. Plant Mol. Biol. 29, 37–51. doi: 10.1007/BF00019117 7579166

[B27] Dar F. A. Mushtaq N. U. Saleem S. Rehman R. U. Dar T. U. H. Hakeem K. R. (2022). Role of epigenetics in modulating phenotypic plasticity against abiotic stresses in plants. Int. J. Genomics 2022, 13. doi: 10.1155/2022/1092894 PMC9213152 35747076

[B28] Ding X. Guo J. Zhang Q. Yu L. Zhao T. Yang S. (2021). Heat-responsive miRNAs participate in the regulation of male fertility stability in soybean CMS-based F1 under high temperature stress. Int. J. Mol. Sci. 22, 2446. doi: 10.3390/ijms22052446 33671046 PMC7957588

[B29] Domingos P. Prado A. M. Wong A. Gehring C. Feijo J. A. (2015). Nitric oxide: a multitasked signaling gas in plants. Mol. Plant 8, 506–520. doi: 10.1016/j.molp.2014.12.010 25680232

[B30] Döring P. Treuter E. Kistner C. Lyck R. Chen A. Nover L. (2000). The role of AHA motifs in the activator function of tomato heat stress transcription factors HsfA1 and HsfA2. Plant Cell 12, 265–278. doi: 10.1105/tpc.12.2.265 10662862 PMC139763

[B31] Driedonks N. Rieu I. Vriezen W. H. (2016). Breeding for plant heat tolerance at vegetative and reproductive stages. Plant Reprod. 29, 67–79. doi: 10.1007/s00497-016-0275-9 26874710 PMC4909801

[B32] Eberharter A. Becker P. B. (2002). Histone acetylation: a switch between repressive and permissive chromatin. EMBO Rep. 3, 224–229. doi: 10.1093/embo-reports/kvf053 11882541 PMC1084017

[B33] Erkina T. Y. Tschetter P. A. Erkine A. M. (2008). Different requirements of the SWI/SNF complex for robust nucleosome displacement at promoters of heat shock factor and Msn2-and Msn4-regulated heat shock genes. Mol. Cell. Biol. 28, 1207–1217. doi: 10.1128/MCB.01069-07 18070923 PMC2258741

[B34] Erkina T. Y. Zou Y. Freeling S. Vorobyev V. Erkine A. M. (2010). Functional interplay between chromatin remodeling complexes RSC, SWI/SNF and ISWI in regulation of yeast heat shock genes. Nucleic Acids Res. 38, 1441–1449. doi: 10.1093/nar/gkp1130 20015969 PMC2836563

[B35] Fahad S. Bajwa A. A. Nazir U. Anjum S. A. Farooq A. Zohaib A.. (2017). Crop production under drought and heat stress: Plant responses and management options. Front. Plant Sci. 8. doi: 10.3389/fpls.2017.01147 PMC5489704 28706531

[B36] FAO. (2022). “Agricultural production statistics. 2000–2020,” in FAOSTAT analytical brief series no. 41. Rome, Italy. ISSN 2709-0078 [Online]

[B37] Fragkostefanakis S. Roeth S. Schleiff E. Scharf K. D. (2015). Prospects of engineering thermotolerance in crops through modulation of heat stress transcription factor and heat shock protein networks. Plant. Cell Environ. 38, 1881–1895. doi: 10.1111/pce.12396 24995670

[B38] Frank G. Pressman E. Ophir R. Althan L. Shaked R. Freedman M.. (2009a). Transcriptional profiling of maturing tomato (*Solanum lycopersicum* l.) microspores reveals the involvement of heat shock proteins, ROS scavengers, hormones, and sugars in the heat stress response. J. Exp. Bot. 60, 3891–3908. doi: 10.1093/jxb/erp234 19628571 PMC2736902

[B39] Frank G. Pressman E. Ophir R. Althan L. Shaked R. Freedman M.. (2009b). Transcriptional profiling of maturing tomato microspores reveals the involvement of heat shock proteins, ROS scavengers, hormones, and sugars in the heat stress response. J. Exp. Bot. 60, 3891–3908. doi: 10.1093/jxb/erp234 19628571 PMC2736902

[B40] Füßl M. Lassowskat I. Née G. Koskela M. M. Brünje A. Tilak P.. (2018). Beyond histones: new substrate proteins of lysine deacetylases in arabidopsis nuclei. Front. Plant Sci. 9, 461. doi: 10.3389/fpls.2018.00461 29692793 PMC5902713

[B41] Ghildiyal M. Zamore P. D. (2009). Small silencing RNAs: an expanding universe. Nat. Rev. Genet. 10, 94–108. doi: 10.1038/nrg2504 19148191 PMC2724769

[B42] González R. M. Ricardi M. M. Iusem N. D. (2013). Epigenetic marks in an adaptive water stress-responsive gene in tomato roots under normal and drought conditions. Epigenetics 8, 864–872. doi: 10.4161/epi.25524 23807313 PMC3883789

[B43] Gou M. Hua J. (2012). Complex regulation of an r gene SNC1 revealed by autoimmune mutants. Plant Signaling Behav. 7, 213–216. doi: 10.4161/psb.18884 PMC3405709 22415045

[B44] Gratani L. (2014). Plant phenotypic plasticity in response to environmental factors. Adv. Bot. 2014, 17. doi: 10.1155/2014/208747

[B45] Gray W. M. Östin A. Sandberg G. Romano C. P. Estelle M. (1998). High temperature promotes auxin-mediated hypocotyl elongation in arabidopsis. Proc. Natl. Acad. Sci. 95, 7197–7202. doi: 10.1073/pnas.95.12.7197 9618562 PMC22781

[B46] Grover A. Mittal D. Negi M. Lavania D. (2013). Generating high temperature tolerant transgenic plants: Achievements and challenges. Plant Sci. 205-206, 38–47. doi: 10.1016/j.plantsci.2013.01.005 23498861

[B47] Guo M. Lu J.-P. Zhai Y.-F. Chai W.-G. Gong Z.-H. Lu M.-H. (2015). Genome-wide analysis, expression profile of heat shock factor gene family (CaHsfs) and characterisation of CaHsfA2 in pepper (Capsicum annuum l.). BMC Plant Biol. 15, 1–20. doi: 10.1186/s12870-015-0512-7 26088319 PMC4472255

[B48] Guo J. Wu J. Ji Q. Wang C. Luo L. Yuan Y.. (2008). Genome-wide analysis of heat shock transcription factor families in rice and arabidopsis. J. Genet. Genomics 35, 105–118. doi: 10.1016/S1673-8527(08)60016-8 18407058

[B49] Hahn A. Bublak D. Schleiff E. Scharf K.-D. (2011). Crosstalk between Hsp90 and Hsp70 chaperones and heat stress transcription factors in tomato. Plant Cell 23, 741–755. doi: 10.1105/tpc.110.076018 21307284 PMC3077788

[B50] Haider S. Iqbal J. Naseer S. Shaukat M. Abbasi B. A. Yaseen T.. (2022). Unfolding molecular switches in plant heat stress resistance: A comprehensive review. Plant Cell Rep. 41, 775–798. doi: 10.1007/s00299-021-02754-w 34401950

[B51] Hasanuzzaman M. Nahar K. Alam M. Roychowdhury R. Fujita M. (2013). Physiological, biochemical, and molecular mechanisms of heat stress tolerance in plants. Int. J. Mol. Sci. 14, 9643–9684. doi: 10.3390/ijms14059643 23644891 PMC3676804

[B52] Hayes S. Schachtschabel J. Mishkind M. Munnik T. Arisz S. A. (2021). Hot topic: Thermosensing in plants. Plant. Cell Environ. 44, 2018–2033. doi: 10.1111/pce.13979 33314270 PMC8358962

[B53] Heerklotz D. DoüRing P. Bonzelius F. Winkelhaus S. Nover L. (2001). The balance of nuclear import and export determines the intracellular distribution and function of tomato heat stress transcription factor HsfA2. Mol. Cell. Biol. 21, 1759–1768 doi: 10.1128/MCB.21.5.1759-1768.2001 11238913 PMC86729

[B54] He J. Jiang Z. Gao L. You C. Ma X. Wang X.. (2019). Genome-wide transcript and small RNA profiling reveals transcriptomic responses to heat stress. Plant Physiol. 181, 609–629. doi: 10.1104/pp.19.00403 31395615 PMC6776850

[B55] Hirayama T. Shinozaki K. (2010). Research on plant abiotic stress responses in the post-genome era: past, present and future. Plant J. 61, 1041–1052. doi: 10.1111/j.1365-313X.2010.04124.x 20409277

[B56] Hsu P. Y. Harmer S. L. (2014). Wheels within wheels: the plant circadian system. Trends Plant Sci. 19, 240–249. doi: 10.1016/j.tplants.2013.11.007 24373845 PMC3976767

[B57] Hua J. (2013). Modulation of plant immunity by light, circadian rhythm, and temperature. Curr. Opin. Plant Biol. 16, 406–413. doi: 10.1016/j.pbi.2013.06.017 23856082

[B58] Ito H. Gaubert H. Bucher E. Mirouze M. Vaillant I. Paszkowski J. (2011). An siRNA pathway prevents transgenerational retrotransposition in plants subjected to stress. Nature 472, 115–119. doi: 10.1038/nature09861 21399627

[B59] Iwasaki M. Paszkowski J. (2014). Identification of genes preventing transgenerational transmission of stress-induced epigenetic states. Proc. Natl. Acad. Sci. 111, 8547–8552. doi: 10.1073/pnas.1402275111 24912148 PMC4060648

[B60] Janni M. Gullì M. Maestri E. Marmiroli M. Valliyodan B. Nguyen H. T.. (2020). Molecular and genetic bases of heat stress responses in crop plants and breeding for increased resilience and productivity. J. Exp. Bot. 71, 3780–3802. doi: 10.1093/jxb/eraa034 31970395 PMC7316970

[B61] Jiang Y. Huang B. (2001). Drought and heat stress injury to two cool-season turfgrasses in relation to antioxidant metabolism and lipid peroxidation. Crop Sci. 41, 436–442. doi: 10.2135/cropsci2001.412436x

[B62] Kakani V. Reddy K. Koti S. Wallace T. Prasad P. Reddy V.. (2005). Differences in *in vitro* pollen germination and pollen tube growth of cotton cultivars in response to high temperature. Ann. Bot. 96, 59–67. doi: 10.1093/aob/mci149 15851397 PMC4246808

[B63] Karkute S. G. Krishna R. Ansari W. A. Singh B. Singh P. M. Singh M. Singh A. K. (2019). Heterologous expression of the AtDREB1A gene in tomato confers tolerance to chilling stress. Biol Plant. 63 (1), 268–277. doi: 10.32615/bp.2019.031

[B64] Kashyap S. Prasanna H. Kumari N. Mishra P. Singh B. (2020). Understanding salt tolerance mechanism using transcriptome profiling and *de novo* assembly of wild tomato solanum chilense. Sci. Rep. 10, 1–20. doi: 10.1038/s41598-020-72474-w 32985535 PMC7523002

[B65] Keller M. Hu Y. Mesihovic A. Fragkostefanakis S. Schleiff E. Simm S. (2017). Alternative splicing in tomato pollen in response to heat stress. DNA Res. 24, 205–217. doi: 10.1093/dnares/dsw051 28025318 PMC5397606

[B66] Kilian B. Dempewolf H. Guarino L. Werner P. Coyne C. Warburton M. L. (2021). Crop science special issue: Adapting agriculture to climate change: A walk on the wild side. Crop Sci. 61, 32–36. doi: 10.1002/csc2.20418

[B67] Knapp S. (2002). Tobacco to tomatoes: a phylogenetic perspective on fruit diversity in the solanaceae. J. Exp. Bot. 53, 2001–2022. doi: 10.1093/jxb/erf068 12324525

[B68] Koini M. A. Alvey L. Allen T. Tilley C. A. Harberd N. P. Whitelam G. C.. (2009). High temperature-mediated adaptations in plant architecture require the bHLH transcription factor PIF4. Curr. Biol. 19, 408–413. doi: 10.1016/j.cub.2009.01.046 19249207

[B69] Kosová K. Vítámvás P. Prášil I. T. Renaut J. (2011). Plant proteome changes under abiotic stress — contribution of proteomics studies to understanding plant stress response. J. Proteomics 74, 1301–1322. doi: 10.1016/j.jprot.2011.02.006 21329772

[B70] Kotak S. Larkindale J. Lee U. Von Koskull-Döring P. Vierling E. Scharf K.-D. (2007). Complexity of the heat stress response in plants. Curr. Opin. Plant Biol. 10, 310–316. doi: 10.1016/j.pbi.2007.04.011 17482504

[B71] Kouhi F. Sorkheh K. Ercisli S. (2020). MicroRNA expression patterns unveil differential expression of conserved miRNAs and target genes against abiotic stress in safflower. PloS One 15, e0228850. doi: 10.1371/journal.pone.0228850 32069300 PMC7028267

[B72] Krasensky J. Jonak C. (2012). Drought, salt, and temperature stress-induced metabolic rearrangements and regulatory networks. J. Exp. Bot. 63, 1593–1608. doi: 10.1093/jxb/err460 22291134 PMC4359903

[B73] Kumar V. Thakur J. K. Prasad M. (2021). Histone acetylation dynamics regulating plant development and stress responses. Cell. Mol. Life Sci. 78, 4467–4486. doi: 10.1007/s00018-021-03794-x 33638653 PMC11072255

[B74] Kumar S. V. Wigge P. A. (2010). H2A. z-containing nucleosomes mediate the thermosensory response in arabidopsis. Cell 140, 136–147. doi: 10.1016/j.cell.2009.11.006 20079334

[B75] Larkindale J. Vierling E. (2008). Core genome responses involved in acclimation to high temperature. Plant Physiol. 146, 748. doi: 10.1104/pp.107.112060 18055584 PMC2245833

[B76] Lee H. Yoo S. J. Lee J. H. Kim W. Yoo S. K. Fitzgerald H.. (2010). Genetic framework for flowering-time regulation by ambient temperature-responsive miRNAs in arabidopsis. Nucleic Acids Res. 38, 3081–3093. doi: 10.1093/nar/gkp1240 20110261 PMC2875011

[B77] Lee J. H. Yoo S. J. Park S. H. Hwang I. Lee J. S. Ahn J. H. (2007). Role of SVP in the control of flowering time by ambient temperature in arabidopsis. Genes Dev. 21, 397–402. doi: 10.1101/gad.1518407 17322399 PMC1804328

[B78] Li B. Carey M. Workman J. L. (2007). The role of chromatin during transcription. Cell 128, 707–719. doi: 10.1016/j.cell.2007.01.015 17320508

[B79] Li J. Huang Q. Sun M. Zhang T. Li H. Chen B.. (2016). Global DNA methylation variations after short-term heat shock treatment in cultured microspores of brassica napus cv. topas. Sci. Rep. 6, 1–13. doi: 10.1038/srep38401 27917903 PMC5137020

[B80] Li Z. Jiang G. Liu X. Ding X. Zhang D. Wang X.. (2020). Histone demethylase SlJMJ6 promotes fruit ripening by removing H3K27 methylation of ripening-related genes in tomato. New Phytol. 227, 1138–1156. doi: 10.1111/nph.16590 32255501

[B81] Li S. Liu J. Liu Z. Li X. Wu F. He Y. (2014). Heat-induced tas1 target1 mediates thermotolerance *via* heat stress transcription factor A1a–directed pathways in arabidopsis. Plant Cell 26, 1764–1780. doi: 10.1105/tpc.114.124883 24728648 PMC4036584

[B82] Lin Y.-X. Jiang H.-Y. Chu Z.-X. Tang X.-L. Zhu S.-W. Cheng B.-J. (2011). Genome-wide identification, classification and analysis of heat shock transcription factor family in maize. BMC Genomics 12, 1–14. doi: 10.1186/1471-2164-12-76 PMC3039612 21272351

[B83] Lippmann R. Babben S. Menger A. Delker C. Quint M. (2019). Development of wild and cultivated plants under global warming conditions. Curr. Biol. 29, R1326–R1338. doi: 10.1016/j.cub.2019.10.016 31846685

[B84] Liu J. Feng L. Gu X. Deng X. Qiu Q. Li Q.. (2019). An H3K27me3 demethylase-HSFA2 regulatory loop orchestrates transgenerational thermomemory in arabidopsis. Cell Res. 29, 379–390. doi: 10.1038/s41422-019-0145-8 30778176 PMC6796840

[B85] Liu J. Feng L. Li J. He Z. (2015). Genetic and epigenetic control of plant heat responses. Front. Plant Sci. 6, 267. doi: 10.3389/fpls.2015.00267 25964789 PMC4408840

[B86] Liu J. X. Howell S. H. (2016). Managing the protein folding demands in the endoplasmic reticulum of plants. New Phytol. 211, 418–428. doi: 10.1111/nph.13915 26990454

[B87] Liu H.-C. Liao H.-T. Charng Y.-Y. (2011). The role of class A1 heat shock factors (HSFA1s) in response to heat and other stresses in arabidopsis. Plant. Cell Environ. 34, 738–751. doi: 10.1111/j.1365-3040.2011.02278.x 21241330

[B88] López-Galiano M. J. González-Hernández A. I. Crespo-Salvador O. Rausell C. Real M. D. Escamilla M.. (2018). Epigenetic regulation of the expression of WRKY75 transcription factor in response to biotic and abiotic stresses in solanaceae plants. Plant Cell Rep. 37, 167–176. doi: 10.1007/s00299-017-2219-8 29079899

[B89] Luo Q. (2011). Temperature thresholds and crop production: a review. Clim. Change 109, 583–598. doi: 10.1007/s10584-011-0028-6

[B90] Martin G. B. Bogdanove A. J. Sessa G. (2003). Understanding the functions of plant disease resistance proteins. Annu. Rev. Plant Biol. 54, 23–61. doi: 10.1146/annurev.arplant.54.031902.135035 14502984

[B91] Matsuoka D. Soga K. Yasufuku T. Nanmori T. (2018). Control of plant growth and development by overexpressing MAP3K17, an ABA-inducible MAP3K, in arabidopsis. Plant Biotechnol. 18, 0412 a. doi: 10.5511/plantbiotechnology.18.0412a PMC6879389 31819720

[B92] Matthews C. Arshad M. Hannoufa A. (2019). Alfalfa response to heat stress is modulated by microRNA156. Physiol. plantarum. 165, 830–842. doi: 10.1111/ppl.12787 29923601

[B93] Ma J. Zhao P. Liu S. Yang Q. Guo H. (2020). The control of developmental phase transitions by microRNAs and their targets in seed plants. Int. J. Mol. Sci. 21 (6), 1971. doi: 10.3390/ijms21061971 PMC7139601 32183075

[B94] Mishra S. K. Tripp J. Winkelhaus S. Tschiersch B. Theres K. Nover L.. (2002). In the complex family of heat stress transcription factors, HsfA1 has a unique role as master regulator of thermotolerance in tomato. Genes Dev. 16, 1555–1567. doi: 10.1101/gad.228802 12080093 PMC186353

[B95] Mlynárová L. Nap J. P. Bisseling T. (2007). The SWI/SNF chromatin-remodeling gene AtCHR12 mediates temporary growth arrest in arabidopsis thaliana upon perceiving environmental stress. Plant J. 51, 874–885. doi: 10.1111/j.1365-313X.2007.03185.x 17605754

[B96] Morrison M. J. Stewart D. W. (2002). Heat stress during flowering in summer brassica. Crop Sci. 42, 797–803. doi: 10.2135/cropsci2002.7970

[B97] Nagel D. H. Pruneda-Paz J. L. Kay S. A. (2014). FBH1 affects warm temperature responses in the arabidopsis circadian clock. Proc. Natl. Acad. Sci. 111, 14595–14600. doi: 10.1073/pnas.1416666111 25246594 PMC4210019

[B98] Nievola C. C. Carvalho C. P. Carvalho V. Rodrigues E. (2017). Rapid responses of plants to temperature changes. Temp. (Austin Tex.) 4, 371–405. doi: 10.1080/23328940.2017.1377812 PMC5800372 29435478

[B99] Ohama N. Sato H. Shinozaki K. Yamaguchi-Shinozaki K. (2017). Transcriptional regulatory network of plant heat stress response. Trends Plant Sci. 22, 53–65. doi: 10.1016/j.tplants.2016.08.015 27666516

[B100] Pandey G. Sharma N. Pankaj Sahu P. Prasad M. (2016). Chromatin-based epigenetic regulation of plant abiotic stress response. Curr. Genomics 17, 490–498. doi: 10.2174/1389202917666160520103914 28217005 PMC5282600

[B101] Pan C. Ye L. Zheng Y. Wang Y. Yang D. Liu X.. (2017). Identification and expression profiling of microRNAs involved in the stigma exsertion under high-temperature stress in tomato. BMC Genomics 18, 843. doi: 10.1186/s12864-017-4238-9 29096602 PMC5668977

[B102] Papikian A. Liu W. Gallego-Bartolomé J. Jacobsen S. E. (2019). Site-specific manipulation of arabidopsis loci using CRISPR-Cas9 SunTag systems. Nat. Commun. 10, 1–11. doi: 10.1038/s41467-019-08736-7 30760722 PMC6374409

[B103] Peralta I. E. Knapp S. Spooner D. M. (2006). Nomenclature for wild and cultivated tomatoes. Tomato Genet. Coop. Rep. 56, 6–12.

[B104] Pickar-Oliver A. Gersbach C. A. (2019). The next generation of CRISPR–cas technologies and applications. Nat. Rev. Mol. Cell Biol. 20, 490–507. doi: 10.1038/s41580-019-0131-5 31147612 PMC7079207

[B105] Popova O. V. Dinh H. Q. Aufsatz W. Jonak C. (2013). The RdDM pathway is required for basal heat tolerance in arabidopsis. Mol. Plant 6, 396–410. doi: 10.1093/mp/sst023 23376771 PMC3603006

[B106] Portoles S. Mas P. (2010). The functional interplay between protein kinase CK2 and CCA1 transcriptional activity is essential for clock temperature compensation in arabidopsis. PloS Genet. 6, e1001201. doi: 10.1371/journal.pgen.1001201 21079791 PMC2973838

[B107] Prasad P. V. Bheemanahalli R. Jagadish S. K. (2017). Field crops and the fear of heat stress–opportunities, challenges and future directions. Field Crops Res. 200, 114–121. doi: 10.1016/j.fcr.2016.09.024

[B108] Qu A.-L. Ding Y.-F. Jiang Q. Zhu C. (2013). Molecular mechanisms of the plant heat stress response. Biochem. Biophys. Res. Commun. 432 (2), 203-207. doi: 10.1016/j.bbrc.2013.01.104 23395681

[B109] Raja M. M. Vijayalakshmi G. Naik M. L. Basha P. O. Sergeant K. Hausman J. F.. (2019). Pollen development and function under heat stress: from effects to responses. Acta Physiol. Plantarum. 41, 1–20. doi: 10.1007/s11738-019-2835-8

[B110] Raza A. Tabassum J. Kudapa H. Varshney R. K. (2021). Can omics deliver temperature resilient ready-to-grow crops? Crit. Rev. Biotechnol. 41, 1209–1232. doi: 10.1080/07388551.2021.1898332 33827346

[B111] Ritossa F. (1962). A new puffing pattern induced by temperature shock and DNP in drosophila. Experientia 18, 571–573. doi: 10.1007/BF02172188

[B112] Rogers K. Chen X. (2013). Biogenesis, turnover, and mode of action of plant microRNAs. Plant Cell 25, 2383–2399. doi: 10.1105/tpc.113.113159 23881412 PMC3753372

[B113] Sailaja B. Voleti S. Subrahmanyam D. Sarla N. Prasanth V. V. Bhadana V.. (2014). Prediction and expression analysis of miRNAs associated with heat stress in oryza sativa. Rice Sci. 21, 3–12. doi: 10.1016/S1672-6308(13)60164-X

[B114] Saraswat S. Yadav A. K. Sirohi P. Singh N. K. (2017). Role of epigenetics in crop improvement: water and heat stress. J. Plant Biol. 60, 231–240. doi: 10.1007/s12374-017-0053-8

[B115] Scharf K.-D. Berberich T. Ebersberger I. Nover L. (2012). The plant heat stress transcription factor (Hsf) family: structure, function and evolution. Biochim. Biophys. Acta (BBA)-Gene Regul. Mech. 1819, 104–119. doi: 10.1016/j.bbagrm.2011.10.002 22033015

[B116] Scharf K.-D. Höhfeld I. Nover L. (1998). Heat stress response and heat stress transcription factors. J. Biosci. 23, 313–329. doi: 10.1007/BF02936124

[B117] Scharf K.-D. Rose S. Zott W. Schöffl F. Nover L. Schöff F. (1990). Three tomato genes code for heat stress transcription factors with a region of remarkable homology to the DNA-binding domain of the yeast HSF. EMBO J. 9, 4495–4501. doi: 10.1002/j.1460-2075.1990.tb07900.x 2148291 PMC552242

[B118] Serrano I. Romero-Puertas M. C. Sandalio L. M. Olmedilla A. (2015). The role of reactive oxygen species and nitric oxide in programmed cell death associated with self-incompatibility. J. Exp. Bot. 66, 2869–2876. doi: 10.1093/jxb/erv083 25750430

[B119] Shi X. Jiang F. Wen J. Wu Z. (2019). Overexpression of solanum habrochaites microRNA319d (sha-miR319d) confers chilling and heat stress tolerance in tomato (S. lycopersicum). BMC Plant Biol. 19, 1–17. doi: 10.1186/s12870-019-1823-x 31122194 PMC6533698

[B120] Stavang J. A. Gallego-Bartolomé J. Gómez M. D. Yoshida S. Asami T. Olsen J. E.. (2009). Hormonal regulation of temperature-induced growth in arabidopsis. Plant J. 60, 589–601. doi: 10.1111/j.1365-313X.2009.03983.x 19686536

[B121] Stief A. Altmann S. Hoffmann K. Pant B. D. Scheible W.-R. Bäurle I. (2014). Arabidopsis miR156 regulates tolerance to recurring environmental stress through SPL transcription factors. Plant Cell 26, 1792–1807. doi: 10.1105/tpc.114.123851 24769482 PMC4036586

[B122] Sun L. Jing Y. Liu X. Li Q. Xue Z. Cheng Z.. (2020). Heat stress-induced transposon activation correlates with 3D chromatin organization rearrangement in arabidopsis. Nat. Commun. 11, 1–13. doi: 10.1038/s41467-020-15809-5 32312999 PMC7170881

[B123] Sun J. Qi L. Li Y. Chu J. Li C. (2012). PIF4–mediated activation of YUCCA8 expression integrates temperature into the auxin pathway in regulating arabidopsis hypocotyl growth. PloS Genet. 8, e1002594. doi: 10.1371/journal.pgen.1002594 22479194 PMC3315464

[B124] Suzuki N. Katano K. (2018). Coordination between ROS regulatory systems and other pathways under heat stress and pathogen attack. Front. Plant Sci. 9. doi: 10.3389/fpls.2018.00490 PMC5911482 29713332

[B125] Taheri S. Gantait S. Azizi P. Mazumdar P. (2022). Drought tolerance improvement in solanum lycopersicum: an insight into “OMICS” approaches and genome editing. 3 Biotech. 12, 1–20. doi: 10.1007/s13205-022-03132-3 PMC8825918 35186660

[B126] Tran M. T. Doan D. T. H. Kim J. Song Y. J. Sung Y. W. Das S.. (2021). CRISPR/Cas9-based precise excision of SlHyPRP1 domain (s) to obtain salt stress-tolerant tomato. Plant Cell Rep. 40, 999–1011. doi: 10.1007/s00299-020-02622-z 33074435

[B127] Traverso J. A. Pulido A. Rodríguez-García M. I. Alché J. D. (2013). Thiol-based redox regulation in sexual plant reproduction: new insights and perspectives. Front. Plant Sci. 4, 465. doi: 10.3389/fpls.2013.00465 24294217 PMC3827552

[B128] Vanderauwera S. Suzuki N. Miller G. Van De Cotte B. Morsa S. Ravanat J.-L.. (2011). Extranuclear protection of chromosomal DNA from oxidative stress. Proc. Natl. Acad. Sci. 108, 1711–1716. doi: 10.1073/pnas.1018359108 21220338 PMC3029710

[B129] Wang J. Li G. Li C. Zhang C. Cui L. Ai G.. (2021). NF-y plays essential roles in flavonoid biosynthesis by modulating histone modifications in tomato. New Phytol. 229, 3237–3252. doi: 10.1111/nph.17112 33247457

[B130] Xie K. Yang Y. (2013). RNA-Guided genome editing in plants using a CRISPR–cas system. Mol. Plant 6, 1975–1983. doi: 10.1093/mp/sst119 23956122

[B131] Xue G.-P. Sadat S. Drenth J. Mcintyre C. L. (2014). The heat shock factor family from triticum aestivum in response to heat and other major abiotic stresses and their role in regulation of heat shock protein genes. J. Exp. Bot. 65, 539–557. doi: 10.1093/jxb/ert399 24323502 PMC3904712

[B132] Yang S. Hua J. (2004). A haplotype-specific resistance gene regulated by BONZAI1 mediates temperature-dependent growth control in arabidopsis. Plant Cell 16, 1060–1071. doi: 10.1105/tpc.020479 15031411 PMC412877

[B133] Yang X. Zhu W. Zhang H. Liu N. Tian S. (2016). Heat shock factors in tomatoes: genome-wide identification, phylogenetic analysis and expression profiling under development and heat stress. PeerJ 4, e1961–e1961. doi: 10.7717/peerj.1961 27190703 PMC4867723

[B134] Young L. W. Wilen R. W. Bonham-Smith P. C. (2004). High temperature stress of brassica napus during flowering reduces micro-and megagametophyte fertility, induces fruit abortion, and disrupts seed production. J. Exp. Bot. 55, 485–495. doi: 10.1093/jxb/erh038 14739270

[B135] Yue H. Nie X. Yan Z. Weining S. (2019). N6-methyladenosine regulatory machinery in plants: composition, function and evolution. Plant Biotechnol. J. 17, 1194–1208. doi: 10.1111/pbi.13149 31070865 PMC6576107

[B136] Yu X. Gao Q. Chen G. Guo J.-E. Guo X. Tang B.. (2018). SlHDA5, a tomato histone deacetylase gene, is involved in responding to salt, drought, and ABA. Plant Mol. Biol. Rep. 36, 36–44. doi: 10.1007/s11105-017-1057-8

[B137] Yu X. Wang H. Lu Y. De Ruiter M. Cariaso M. Prins M.. (2012). Identification of conserved and novel microRNAs that are responsive to heat stress in brassica rapa. J. Exp. Bot. 63, 1025–1038. doi: 10.1093/jxb/err337 22025521 PMC3254694

[B138] Yu W. Wang L. Zhao R. Sheng J. Zhang S. Li R.. (2019). Knockout of SlMAPK3 enhances tolerance to heat stress involving ROS homeostasis in tomato plants. BMC Plant Biol. 19, 1–13. doi: 10.1186/s12870-019-1939-z 31412779 PMC6694692

[B139] Zhang C. Li G. Chen T. Feng B. Fu W. Yan J.. (2018). Heat stress induces spikelet sterility in rice at anthesis through inhibition of pollen tube elongation interfering with auxin homeostasis in pollinated pistils. Rice 11, 1–12. doi: 10.1186/s12284-018-0206-5 29532187 PMC5847639

[B140] Zhao J. Lu Z. Wang L. Jin B. (2020). Plant responses to heat stress: physiology, transcription, noncoding RNAs, and epigenetics. Int. J. Mol. Sci. 22, 117. doi: 10.3390/ijms22010117 PMC7795586 33374376

[B141] Zheng H.-X. Sun X. Zhang X.-S. Sui N. (2020). m6A editing: new tool to improve crop quality? Trends Plant Sci. 25, 859–867. doi: 10.1016/j.tplants.2020.04.005 32376086

[B142] Zhou L. Gao G. Tang R. Wang W. Wang Y. Tian S.. (2022a). m6A-mediated regulation of crop development and stress responses. Plant Biotechnol. J 20, 156. doi: 10.1111/pbi.13792 PMC9342612 35178842

[B143] Zhou R. Jiang F. Niu L. Song X. Yu L. Yang Y.. (2022b). Increase crop resilience to heat stress using omic strategies. Front. Plant Sci. 13. doi: 10.3389/fpls.2022.891861 PMC9152541 35656008

[B144] Zhou R. Yu X. Ottosen C.-O. Zhang T. Wu Z. Zhao T. (2020). Unique miRNAs and their targets in tomato leaf responding to combined drought and heat stress. BMC Plant Biol. 20, 107. doi: 10.1186/s12870-020-2313-x 32143575 PMC7060562

[B145] Zhuang L. Cao W. Wang J. Yu J. Yang Z. Huang B. (2018). Characterization and functional analysis of FaHsfC1b from festuca arundinacea conferring heat tolerance in arabidopsis. Int. J. Mol. Sci. 19, 2702. doi: 10.3390/ijms19092702 PMC6163916 30208588

[B146] Zhu Y. Qian W. Hua J. (2010). Temperature modulates plant defense responses through NB-LRR proteins. PloS Pathog. 6, e1000844. doi: 10.1371/journal.ppat.1000844 20368979 PMC2848567

[B147] Zilberman D. Gehring M. Tran R. K. Ballinger T. Henikoff S. (2007). Genome-wide analysis of arabidopsis thaliana DNA methylation uncovers an interdependence between methylation and transcription. Nat. Genet. 39, 61–69. doi: 10.1038/ng1929 17128275

[B148] Zinn K. E. Tunc-Ozdemir M. Harper J. F. (2010). Temperature stress and plant sexual reproduction: uncovering the weakest links. J. Exp. Bot. 61, 1959–1968. doi: 10.1093/jxb/erq053 20351019 PMC2917059

